# Multiferroic and Phonon Properties of the Double Perovskite Pr_2_FeAlO_6_

**DOI:** 10.3390/ma17194785

**Published:** 2024-09-29

**Authors:** Angel T. Apostolov, Iliana N. Apostolova, Julia M. Wesselinowa

**Affiliations:** 1 Department of Physics, Faculty of Hydrotechnics, University of Architecture, Civil Engineering and Geodesy, Hristo Smirnenski Blvd. 1, 1046 Sofia, Bulgaria; angelapos@abv.bg; 2 Faculty of Forest Industry, University of Forestry, Kl. Ohridsky Blvd. 10, 1756 Sofia, Bulgaria; inaapos@abv.bg; 3 Faculty of Physics, Sofia University “St. Kliment Ohridski”, J. Bouchier Blvd. 5, 1164 Sofia, Bulgaria

**Keywords:** Pr_2_FeAlO_6_, magnetoelectric effect, phonon energy, microscopic model, Green’s function theory

## Abstract

With the help of a microscopic model and Green’s function technique, we studied the multiferroic and phonon properties of the recently reported new multiferroic Pr_2_FeAlO_6_ (PFAO) compound, which belongs to the double perovskite A_2_BB’O_6_ family. The magnetization decreases with the increase in temperature and disappears at the ferromagnetic Curie temperature $T_{C}^{FM}$. The polarization increases with the application of an external magnetic field, indicating strong magnetoelectric coupling and confirming the multiferroic behavior of PFAO. In the curves of dependence of the phonon energy and their damping with respect to temperature, a kink is observed at $T_{C}^{FM}$. This is due to the strong anharmonic spin–phonon interactions, which play a crucial role below $T_{C}^{FM}$ and are frequently observed in other double perovskite compounds. Above $T_{C}^{FM}$, only anharmonic phonon–phonon coupling remains. The phonon mode is controlled by an external magnetic field.

## 1. Introduction

Multiferroic materials, which possess interacted ferroelectric and ferromagnetic order parameters, are promising candidates for multifunctional devices that can be controlled both electrically and magnetically [1]. However, multiferroics with significant magnetoelectric coupling are relatively rare. Recently, Liu et al. [2] studied the multiferroic and magnetodielectric effects in Pr_2_FeAlO_6_ (PFAO). Its crystal structure is similar to the earlier reported nanopolycrystalline Pr_2_FeCrO_6_ [3]. PFAO is a type I multiferroics and belongs to the family of double perovskites A_2_BB’O_6_, where A is a rare earth element and B/B′ are transition metals. It exhibits significant magnetoresistive and electronic properties, a high Curie temperature, and a strong spin polarization [4,5]. In such materials, electric polarization can be further enhanced by the formation of square bonds between B or B′ ions and through the presence of oxygen vacancies. For example, introducing non-magnetic Al ions into the B-site, as shown in Y_2_FeAlO_6_, increases polarization [6]. Double perovskites feature a doubled unit cell compared with the conventional perovskite structure (ABO_3_), with alternating BO_6_ and B’O_6_ octahedra forming two interpenetrating sublattices. The wide range of possible A and B/B’ ion combinations in double perovskites allows for various configurations that support the coexistence of ferromagnetism and ferroelectricity. Many multiferroic double perovskite compounds, such as La_2_NiMnO_6_, Dy_2_NiMnO_6_, Sm_2_NiMnO_6_, Pr_2_FeCrO_6_, and Y_2_FeAlO_6_, have been investigated, both theoretically and experimentally [7,8,9,10,11]. These compounds exhibit various remarkable properties, suggesting potential applications. The coupling and changing of multiple properties may also enable their use in multifunctional devices that intersect various technological domains [5].

There are not many studies on the phonon properties of these double perovskites. Raman spectroscopy measurements as a function of temperature were performed on Y_2_NiMnO_6_ and La_2_CoMnO_6_ ceramics by Filho et al. [12], Silva et al. [13], and Iliev et al. [14]. The Raman spectra of PFAO and La_2_CoMnO_6_ were observed by Liu et al. [2] and Harbi et al. [15]. Raman studies of La_2_CoMnO_6_ thin film grown on NdGaO_3_ at high temperatures were observed by Kumar et al. [16]. Spin–phonon coupling was detected in Tb_2_NiMnO_6_, although it was found to be weaker than in La_2_NiMnO_6_ and Pr_2_NiMnO_6_ [13,17,18].

In this study, we will explore the electric, magnetic, and phonon properties of the recently discovered multiferroic double perovskite compound PFAO using Green’s function theory and a microscopic model.

## 2. Model and Method

We propose a model to describe the properties of PFAO. As PFAO is a multiferroic compound, the Hamiltonian must include the following three terms for the magnetic system, electrical system, and the one that connects them:

(1) $$H=H_{m}+H_{e}+H_{me}.$$

The Hamiltonian for the magnetic properties, including the spin–phonon coupling, which is observed experimentally in PFAO, can be formulated as follows:

(2) $$H_{m}=H_{sp}+H_{ph}+H_{sp-ph}.$$

The Hamiltonian $H_{sp}$ for the magnetic subsystem is described by the Heisenberg model, which is extended taking into account the super-exchange interactions between Fe and Pr ions:

(3) $$\begin{array}{ccc} H_{sp} & = & -\sum_{i,j}J_{ij}^{Fe-Fe}S_{i}^{Fe}\cdot S_{j}^{Fe}-\sum_{i,j}J_{ij}^{Fe-Pr}S_{i}^{Fe}\cdot S_{j}^{Pr}\\ & - & \sum_{i}D_{i}{\left(S_{i}^{zFe}\right)}^{2}-\sum_{i}D_{i}{\left(S_{i}^{zPr}\right)}^{2}-g\mu_{B}h\cdot \sum_{i}S_{i}^{Fe}. \end{array}$$

The Heisenberg spin operator $S_{i}$ refers to the localized spins at site *i*. The exchange interaction constants $J_{ij}^{Fe-Fe}$ include contributions from both nearest-neighbor interactions $J_{1}>0$ and next-nearest-neighbor interactions $J_{2}<0$ between Fe–Fe ions, and $J_{ij}^{Fe-Pr}>0$ describes the super-exchange interaction of Fe^3+^-O-Pr^3+^. The last interaction is responsible for the ferromagnetic behavior of PFAO due to the much higher anisotropy of the Pr ion compared with that of the Fe ion. The Al^3+^ ion is non-magnetic. The term $D_{i}$ denotes the single-ion anisotropy constant and *h* is the external magnetic field.

The Hamiltonian $H_{ph}$, which is used to study the phonon properties in PFAO, accounts for lattice vibrations, incorporating anharmonic phonon–phonon interactions described by the constants *B* and *A*:

(4) $$\begin{array}{ccc} H_{ph} & = & \frac{1}{2!}\sum_{i}\omega_{0i}a_{i}a_{i}^{+}+\frac{1}{3!}\sum_{i,j,r}B\left(i,j,r\right)Q_{i}Q_{j}Q_{r}\\ & + & \frac{1}{4!}\sum_{i,j,r,s}A\left(i,j,r,s\right)Q_{i}Q_{j}Q_{r}Q_{s}. \end{array}$$

In this formulation, the frequency of the lattice mode is denoted by $\omega_{0i}$, while $Q_{i}$ represents the normal coordinate. $Q_{i}$ can be expressed in terms of the phonon creation operator $a^{+}$ and the annihilation operator *a*:

(5) $$Q_{i}={\left(2\omega_{0i}\right)}^{-1/2}\left(a_{i}+a_{i}^{+}\right).$$

The Hamiltonian describing spin–phonon coupling is expressed as follows:

(6) $$H_{sp-ph}=\frac{1}{2}\sum_{i,j}F\left(i,j\right)Q_{i}S_{j}^{z}-\frac{1}{4}\sum_{i,j,r}R\left(i,j,r\right)Q_{i}Q_{j}S_{r}^{z}+h.c.$$

The amplitudes for the interacting phonons and spin excitations are represented by parameters *F* and *R*, corresponding to first-order and second-order interactions, respectively.

The spontaneous magnetization, $M=\langle S^{z}\rangle$, is calculated using the following equation:

(7) $$M=\langle S^{z}\rangle =\frac{1}{N}\sum_{ij}\left[\left(S+0.5\right)\coth\left[\left(S+0.5\right)\beta E_{mij}\right)]-0.5\coth\left(0.5\beta E_{mij}\right)\right].$$

Here, *S* denotes the spin value, $\beta =1/k_{B}T$, and $E_{mij}$ represents the spin excitations derived from the spin Green’s function $G_{ij}=\ll S_{i}^{+};S_{j}^{-}\gg$ using the method of Tserkovnikov [19]. We will provide a brief description. After a formal integration of the equation of motion for the retarding two-time GF

(8) $$G_{ij}\left(t\right)=\langle \langle S_{i}^{+}\left(t\right);S_{j}^{-}\rangle \rangle$$

one obtains

(9) $$G_{ij}\left(t\right)=-i\theta \left(t\right)\langle \left[S_{i}^{+};S_{j}^{-}\right]\rangle \exp\left(-iE_{ij}\left(t\right)t\right),$$

(10) $$\begin{array}{ccc} E_{ij}\left(t\right)=E_{ij} & - & \frac{i}{t}\int_{0}^{t}dt^{\prime }t^{\prime }\left(\frac{\langle \left[j_{i}\left(t\right);j_{j}^{+}\left(t^{\prime }\right)\right]\rangle }{\langle \left[S_{i}^{+}\left(t\right);S_{j}^{-}\left(t^{\prime }\right)\right]\rangle }\right.\\ & - & \left.\frac{\langle \left[j_{i}\left(t\right);S_{j}^{-}\left(t^{\prime }\right)\right]\rangle \langle \left[S_{i}^{+}\left(t\right);j_{j}^{+}\left(t^{\prime }\right)\right]\rangle }{{\langle \left[S_{i}^{+}\left(t\right);S_{j}^{-}\left(t^{\prime }\right)\right]\rangle }^{2}}\right). \end{array}$$

Here, $j_{i}\left(t\right)=\langle \left[S_{i}^{+}\left(t\right),H_{interaction}\right]\rangle$. $E_{ij}$ is the excitation energy:

(11) $$E_{ij}=\frac{\langle \left[\left[S_{i}^{+},H\right];S_{j}^{-}\right]\rangle }{\langle \left[S_{i}^{+};S_{j}^{-}\right]\rangle }$$

From the time-dependent term in Equation (10) we can calculate the damping effects.

The ferroelectric subsystem is described by the transverse Ising model:

(12) $$H_{e}=-\Omega \sum_{i}B_{i}^{x}-\frac{1}{2}\sum_{ij}J_{ij}^{\prime }B_{i}^{z}B_{j}^{z}.$$

In this context, $B_{i}^{x}$ and $B_{i}^{z}$ denote the pseudo-spin operators, while $J_{ij}^{\prime }$ represents the pseudo-spin exchange interaction. The dynamics of the ferroelectric component are governed by the first term connected with a flipping rate Ω and operator $B^{x}$. A new coordinate system is introduced by rotating the original one used in Equation (12) by an angle θ in the xz plane, such that $\langle B^{x^{\prime }}\rangle =0$ in this rotated system.

The spontaneous polarization *P* is calculated from Green’s function:

(13) $$G_{ij}=\ll B_{i}^{+};B_{j}^{-}\gg$$

as

(14) $$P=\frac{1}{2N^{2}}\sum_{ij}\tanh\frac{E_{fij}}{2k_{B}T}.$$

$E_{fij}$ is the pseudo-spin wave energy.

The term describing the magnetoelectric coupling between the two order parameters is given by the following:

(15) $$H_{me}=-g\sum_{ijkl}B_{i}^{z}B_{j}^{z}S_{k}\cdot S_{l}.$$

We assume a quadratic magnetoelectric interaction in the spin and pseudo-spin operators with a small value for the magnetoelectric constant *g* due to the large difference between the phase transition temperatures of the ferroelectric and ferromagnetic subsystems, $T_{C}^{FE}>>T_{C}^{FM}$, observed in PFAO.

To study the phonon properties, we define Green’s function

(16) $$G_{ij}\left(t\right)=\langle \langle a_{i}\left(t\right);a_{j}^{+}\rangle \rangle .$$

Using Hamiltonian (2), we derive the expression for the phonon energy, which is renormalized by the anharmonic interactions between spin–phonon and phonon–phonon:

(17) $$\begin{array}{ccc} \omega {\left(k\right)}^{2} & = & \omega_{0}^{2}-2\omega_{0}\frac{1}{N^{\prime }}\sum_{q}(R\left(k,q\right){\langle S^{z}\rangle }^{2}\\ & - & \frac{1}{2N^{\prime }}\sum_{q}A\left(k,q\right)\left(2\bar{N}\left(q\right)+1\right)-B\left(k\right)\langle Q_{k}\rangle ), \end{array}$$

with

(18) $$\langle Q_{k}\rangle =\frac{F_{k}{\langle S^{z}\rangle }^{2}-\frac{1}{N^{\prime }}\sum_{q}B_{k,q}\left(2{\bar{N}}_{q}+1\right)}{\omega_{0}-R_{k}{\langle S^{z}\rangle }^{2}+\frac{1}{N^{\prime }}\sum_{q}A_{k,q}\left(2{\bar{N}}_{q}+1\right)}.$$

The phonon correlation function $\bar{N}=\langle a^{+}a^{-}\rangle$ is determined through the Spectral theorem. Additionally, phonon damping is evaluated employing the method of Tserkovnikov [19].

## 3. Numerical Results and Discussion

For the numerical evaluation of the properties of PFAO, the following model parameters are used: $J_{1}^{Fe-Fe}$ = 55 K, $J_{2}^{Fe-Fe}$ = −115 K [20], $J^{Fe-Pr}$ = 47 K, $D^{Fe}$ = 0.2 K, $J^{\prime }$ = 235 K, Ω = 20 K, *g* = 10 K, *F* = 23 cm^−1^, *R* = 18 cm^−1^, *A* = 6.61 cm^−1^, *B* = −2.94 cm^−1^, *S* = 2.5 for the magnetic spins, *S* = 0.5 for the pseudo-spins.

We will provide a short description for some of the observed parameters. From the expression $J=3k_{B}T_{C}/zS\left(S+1\right)$, $T_{C}$ is the calculated interaction constants, *z* denotes the number of nearest neighbors, *S* is the spin value, and $T_{C}$ is the Curie temperature. The value for Ω is observed from the ferroelectric energy at very high temperatures: 2Ω = $E_{f}$.

**Figure 1:**
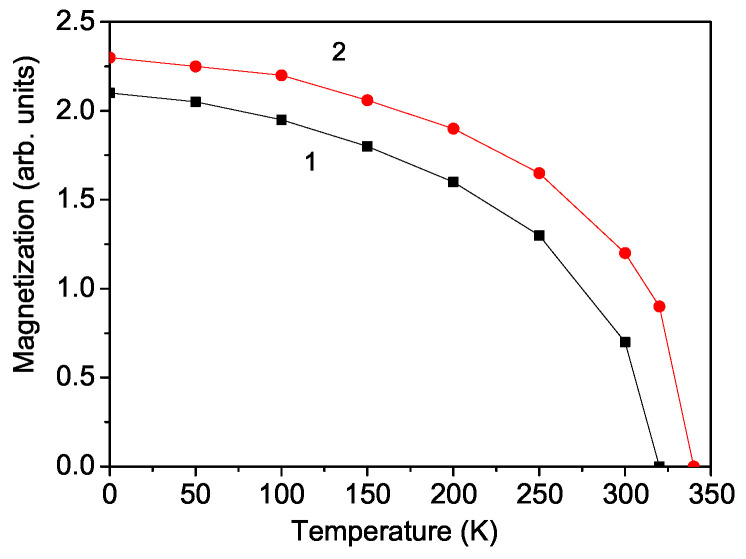
Temperature dependence of the magnetization *M* in PFAO for different *g* values: (1) 10 and (2) 15 K.

**Figure 2:**
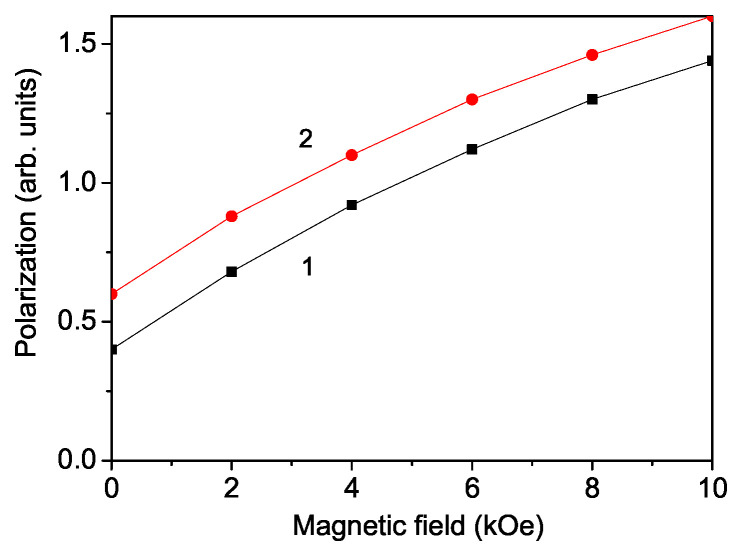
Magnetic field dependence of the polarization *P* in PFAO for different *T* values: (1) 300 and (2) 150 K.

**Figure 3:**
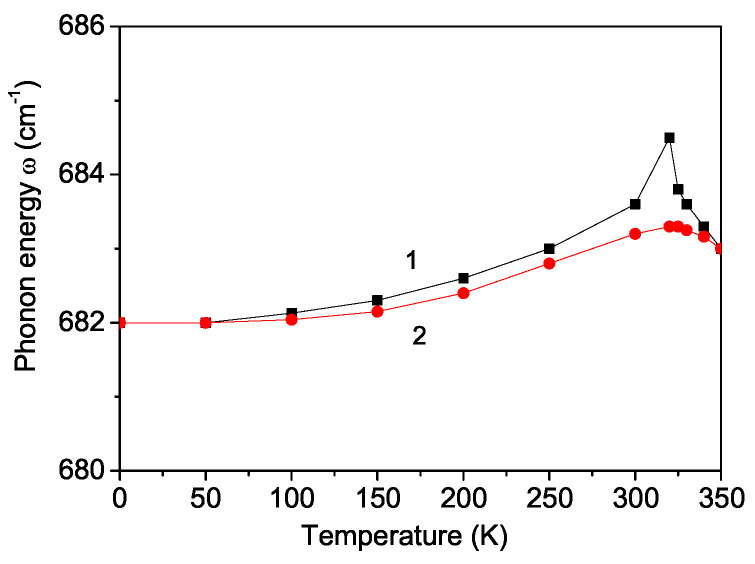
Temperature dependence of the phonon mode ω = 682 cm^−1^ in PFAO for different magnetic fields *h*: (1) 0 and (2) 50 kOe.

**Figure 4:**
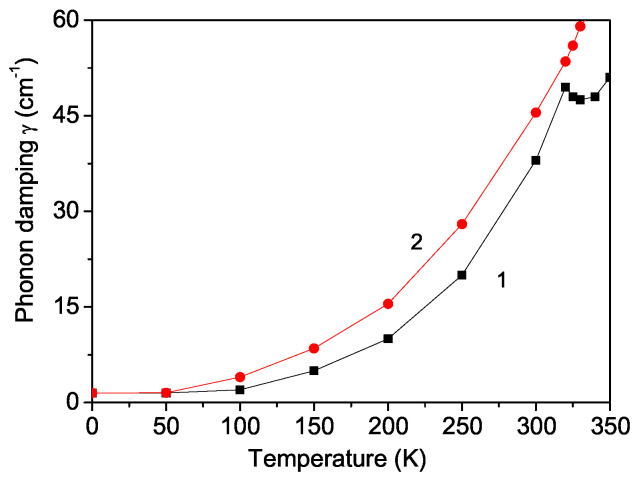
Temperature dependence of the damping of the phonon mode ω = 682 cm^−1^ in PFAO for different magnetic fields *h*: (1) 0 and (2) 50 kOe.

Please note that because this is the first theoretical paper describing the multiferroic properties of PFAO, we only made a qualitative comparison with the first and only experimental work of Liu et al. [2]. Thus, the curves in Figure 1, Figure 2, Figure 3 and Figure 4 are theoretically calculated. The points are the calculated values that are connected through lines.

### 3.1. Temperature Dependence of the Magnetization in PFAO

Firstly, in order to test our model, using Equation (7), we investigated the magnetization *M* of the PFAO compound. It must be noted that the spin–phonon and magnetoelectric interactions lead to a renormalization of the exchange spin-interaction constant *J*, which is modified to $J_{eff}$:

(19) $$J_{eff}=J=2F^{2}/\left(\omega_{0}-MR\right)+2gP^{2}\cos^{2}\theta .$$

Figure 1 presents M(T). It is evident that PFAO demonstrates the ferromagnetic behavior, with *M* decreasing as the temperature rises and reaching zero at $T_{C}^{FM}$ = 320 K. It is the transition temperature from the ferromagnetic to paramagnetic phases, as reported by Liu et al. [2]. This ferromagnetic behavior is primarily attributed to the super-exchange interaction between the Fe and Pr ions (Fe^3+^-O-Pr^3+^). Moreover, magnetization increases with a higher magnetoelectric coupling constant *g* (see Figure 1, curve 2).

### 3.2. Magnetic Field Dependence of the Polarization in PFAO

The polarization is analyzed using Equation (14). A key characteristic of multiferroic materials is the impact of an external magnetic field on their electric properties (and similarly, the influence of an external electric field on their magnetic properties). To explore the multiferroic nature of PFAO, we calculated the magnetic field dependence of the polarization *P*. This behavior arises from the magnetoelectric coupling *g*, which renormalizes the pseudo-spin exchange interaction constant $J^{\prime }$ to $J_{eff}^{\prime }$:

(20) $$J_{eff}^{\prime }=J^{\prime }+2g\left(\langle S^{-}S^{+}\rangle +\langle S^{z}S^{z}\rangle \right)/\cos\theta .$$

The polarization *P* enhances with the increase in the external magnetic field *h*, as is illustrated in Figure 2, which indicates the multiferroic behavior of the double perovskite compound PFAO. In the double perovskite compound under consideration, the structural units responsible for the occurrence of magnetization and polarization are different, as well as the temperatures, and the corresponding phase transitions differ significantly. This determines the nature of the magnetoelectric interaction, which is quadratic in terms of polarization and magnetization. Thus, the effective pseudo-spin arrangement is renormalized by the magnetoelectric coupling, and this renormalization is proportional to ${\langle S^{z}\rangle }^{2}$. The experimentally found and theoretically proven ferromagnetic behavior of the system assumes an increase in magnetization with an increase in the magnetic field *h*. This leads to an increase in $J_{eff}^{\prime }$, which, at a fixed temperature will lead to an increase in polarization with an increase in the magnetic field. This is in disagreement with the result of Liu et al. [2]. They reported a reduction in polarization and dielectric constant of PFAO with an increase in magnetic field *h*. Let us emphasize that we observed an increase in the dielectric constant with an increase in the magnetic field in the double-perovskite La_2_NiMnO_6_ [21]. This increase is confirmed by many experimental data. Additionally, we observed that the polarization *P* increased as the temperature *T* decreased (see Figure 2, curve 2).

### 3.3. Temperature and Magnetic Field Dependence of the Phonon Energy in PFAO

Now, we will investigate the temperature dependence of the A_1g_ phonon mode with a frequency of ω = 682 cm^−1^. Reference [22] calculated the temperature behavior of the phonon energy in hexagonal bulk multiferroics for different *R*, which can be either positive (R>0) or negative (R<0). It was found that for R>0, the phonon energy increased with temperature below $T_{C}^{FM}$, whereas for R<0, it decreased with the rising temperature. For PFAO, we assumed a positive anharmonic spin–phonon constant (R>0). The result is depicted in Figure 3, curve 1. It shows a softening below $T_{C}^{FM}$. It is noteworthy that Filho et al. [12] reported a similar decrease in temperature dependence of the phonon mode ω(T) = 652 cm^−1^ below the ferromagnetic phase transition temperature $T_{C}^{FM}$ = 100 K in the double perovskite Y_2_NiMnO_6_. A similar behavior was noted in several double perovskites (La_2_NiMnO_6_, A_2_CoMnO_6_, where A = La, Pr, Nd) [23,24], respectively, confirming the presence of spin–phonon coupling in these materials. Additionally, a distinct kink in the ω(T) curve at $T_{C}^{FM}$ = 320 K was observed (see Figure 3, curve 1), which indicates a strong spin–phonon interaction *R*. This finding suggests that the stretching vibrational mode of the oxygen octahedra plays a role in stabilizing the magnetic interaction. The combination of ferromagnetic ordering and spin–phonon coupling below $T_{C}^{FM}$ leads to a decrease in the phonon mode with decreasing temperature *T*. Bhatti et al. [25] reported a similar kink at $T_{C}^{FM}$ = 172 K in the temperature dependence of both phonon energy and damping in the double perovskite manganite Pr_2_CoMnO_6_.

Nair et al. [17] proposed a correlation between the size of the rare earth ion and the strength of the spin–phonon coupling, indicating that a decrease in the rare earth ionic radius resulted in reduced spin-phonon coupling. Consequently, they reported that the spin–phonon coupling observed in Tb_2_NiMnO_6_ was weaker than that in La_2_NiMnO_6_ and Pr_2_NiMnO_6_. However, Iliev et al. [14] demonstrated that the strong spin–phonon coupling in Y_2_NiMnO_6_ suggested that rare earth ionic size was not the sole factor influencing the spin–phonon coupling, as this effect occurred in Y_2_NiMnO_6_ despite the smaller ionic radius of Y compared with Tb. We propose that the exchange interactions between the two transition metal ions, as well as those between the transition metal and rare earth ions, are also crucial for determining the strength of the spin–phonon interaction. This topic will be explored in a future publication. Comparing the properties of these materials poses challenges due to their diverse physical characteristics, including various magnetic orderings such as ferromagnetic, antiferromagnetic, or ferrimagnetic [5]. For instance, Bi_2_NiMnO_6_ is a ferromagnet, but La_2_NiMnO_6_ is a ferromagnetic semiconductor. Furthermore, the double perovskites can exhibit ferroelectric, antiferroelectric, or relaxor ferroelectric properties.

The kink shown in Figure 3 is not present when *R* = 0. Below $T_{C}^{FM}$, the temperature dependence of the phonon mode is primarily influenced by spin–phonon interactions, while above $T_{C}^{FM}$, where $\langle S^{z}\rangle$ terms are absent, only anharmonic phonon–phonon interactions remain.

Furthermore, a characteristic of multiferroics is that their properties can be controlled by external fields. Applying an external magnetic field *h* to PFAO causes the phonon energy ω(T) to decrease, which eliminates the kink at $T_{C}^{FM}$. The result is demonstrated in Figure 3, curve 2.

### 3.4. Temperature and Magnetic Field Dependence of Phonon Damping in PFAO

The phonon damping γ, represented by the full width at the half maximum (FWHM) of the Raman peaks, enhances with an increase in temperature. It is important to note that γ rises with *T* for both R>0 and R<0, as it is proportional to $R^{2}$. Figure 4, curve 1, illustrates the temperature dependence of phonon damping without the magnetic field, *h* = 0. γ(T) increases with an increase in temperature *T* and also shows a kink at $T_{C}^{FM}$, although this kink is smaller than that observed in the phonon energy ω(T). The phonon damping γ increases, i.e., the width of the Raman line becomes larger, and the kink disappears with the application of a magnetic field *h* (see Figure 4, curve 2).

Unfortunately, there are currently no experimental data available for ω(T,h) and γ(T,h) in PFAO. We hope that future experimental and theoretical works related to phonon results will confirm our results.

## 4. Conclusions

Using Green’s function technique and a microscopic model, we have examined the multiferroic and phonon properties of the newly discovered multiferroic double perovskite PFAO. The magnetization *M* diminishes as the temperature increases, vanishing at the ferromagnetic Curie temperature $T_{C}^{FM}$. *M* increases with a higher magnetoelectric coupling constant *g*. The application of an external magnetic field enhances the polarization, demonstrating the multiferroic nature of PFAO. The temperature dependence of the phonon mode at ω = 682 cm^−1^ and its damping reveals a kink at $T_{C}^{FM}$, attributed to the anharmonic spin–phonon coupling, which is significant below $T_{C}^{FM}$. Above $T_{C}^{FM}$, only the anharmonic phonon–phonon interactions persist. The application of a magnetic field causes the kinks in both the phonon energy and damping to disappear.

## Data Availability

The raw data supporting the conclusions of this article will be made available by the authors on request.
